# Functional diversity of macrozoobenthos under adverse oxygen conditions in the southern Baltic Sea

**DOI:** 10.1038/s41598-024-59354-3

**Published:** 2024-04-18

**Authors:** Halina Kendzierska, Urszula Janas

**Affiliations:** Department of Marine Ecology, Faculty of Oceanography and Geography, University of Gdańsk, Al. Marszałka Piłsudskiego 46, 81-378 Gdynia, Poland

**Keywords:** Zoology, Ecology, Biodiversity, Ecology, Biodiversity, Community ecology, Ecosystem ecology

## Abstract

Oxygen deficiency is a major problem in the Baltic Sea. To study the impact of hypoxia on the functional diversity of benthic fauna and the possibility of macrozoobenthos recovery, data were analyzed in a gradient of oxygen conditions in the Gdańsk Basin. The research conducted on the basis of biological traits analysis enabled us to analyze the number, type and spatial distribution of biological traits—a proxy for functions performed by macrozoobenthos. A significant depletion of macrofauna was already observed under conditions of reduced oxygen above the bottom, both in terms of functional diversity and biomass. Although taxa observed in hypoxia (DO < 2 mL L^−1^) perform a number of functions, the remaining species do not form complex structures in the sediments or cause deep bioturbation and bioirrigation. Moreover, their extremely low biomass plays an irrelevant role in benthic–pelagic coupling. Thus, benthic fauna under hypoxia is not an element that ensures the functioning of the ecosystem. We assess that traits important for species dispersal and the presence of taxa resistant to short-term hypoxia in the oxic zone above the halocline provide a “backup” for ecosystem functioning under altered diverse oxygen conditions below the halocline after cessation of hypoxia in the southern Baltic Sea.

## Introduction

Oxygen deficiency is a natural or human-induced phenomenon observed in water bodies around the world^1,2^. Some of the largest marine anaerobic areas occur in the Baltic Sea^3,4^. In the deep-water basins of this sea, oxygen deficiencies occur on a long-term basis and are related to the occurrence of strong water stratification and the lack of mixing of well-oxygenated surface waters with bottom waters^5^. They are also the result of perennially high terrigenous nutrient loads and consequently a high influx of organic matter into the sediments^6^. Seasonal or episodic oxygen deficiencies have been recorded in coastal waters^7–11^. Research shows that ongoing climate change contributes to the deterioration of oxygen conditions in seas and oceans^12^. The results of mathematical modeling indicate that future temperature increases will, inter alia, contribute to an increase in the area affected by hypoxia and anoxia in the central Baltic Sea, and that the duration of their impact will be prolonged^13,14^. However, hypoxia can be reduced through further reductions in nutrient loads^14^.

Oxygen depletion affect both the functioning of individual organisms and entire communities, but can also cause changes in ecosystem functioning^15–17^. One of the mechanisms involved in the response to adverse environmental conditions is the escape of mobile fauna from exposed areas^18^. Organisms that are unable to escape respond by changing their behavior, e.g., increasing the rate of ventilation^19^, while infauna emerge to the sediment surface^20,21^. As a result of insufficient levels of dissolved oxygen (DO) in the environment, physiological and biochemical changes are observed in invertebrates, first by maintaining oxygen delivery and transportation (e.g., increasing the respiration rate), then by decreasing the metabolic rate and switching to anaerobic metabolism^15,22,23^. Recurrent hypoxia causes, inter alia, changes in enzyme activity, which results in reduced effectiveness of the immune system^24,25^.

Seasonal episodic hypoxia is known to reduce the number of species, their abundance and biomass^26–28^. Small body size, rapid growth rates, as well as reduced bioturbation and annual life cycles are traits of species inhabiting such zones^29,30^. The presence of such species, also characterized by high tolerance to oxygen depletion and the content of H_2_S, determines the functioning of the bottom of this zone, i.e., the resistance of the ecosystem to hypoxia. There are also species that, while exhibiting low resistance to hypoxia, have characteristics that allow them to quickly colonize the zone when oxygen conditions improve (e.g., mobility). The presence of such species in close proximity to an area exposed to oxygen deficiency enables the ecosystem to recover, i.e., increases its resilience to hypoxia.

Studies of ecological functioning can be based on ecological relations of individual organisms, such as trophic relationships^31,32^, or the ability to modify the environment through animal activity^33,34^. Benthic communities form an interaction matrix of biological traits that drive ecosystem functions (understood as ecosystem processes, i.e., production, decomposition and nutrient cycling) and condition responses to environmental drivers^35^. Therefore, functioning can also be assessed by studying the number, type and spatial distribution of functions performed by organisms in an ecosystem^36^. Biological traits analysis (BTA) is one of the methods used to analyze ecosystem functioning^35,37–39^. The BTA approach uses a number of selected traits of organisms related to their role in the functioning of benthic communities in the ecosystem.

Studies of functional diversity have been conducted in the Baltic Sea both by analyzing individual biological traits and by analyzing the occurrence of functions throughout the ecosystem^38–44^. The impact of oxygen deficiency on the functioning of macrozoobenthos has been analyzed in laboratory studies conducted on individual species^20,21,45^ and, rarely, on communities in experiments^21,46,47^ or on given communities in the environment^48–51^. By collecting data under different oxygen conditions in the Gdańsk Basin, we can fill in the gaps in our knowledge about the transformations of communities under such conditions. The objective of our research was to determine the impact of different oxygen conditions on the structure and, consequently, on the functioning of benthic communities in the southern Baltic Sea. We investigated which functions of the benthic macrofauna in the southern Baltic Sea are constrained by adverse oxygen conditions and which remain represented? And finally, we assessed whether the oxic zone above the halocline provides “backup” for the functioning of the ecosystem below the halocline—a depleted zone under reduced or hypoxic conditions.

## Results

### Bottom–water characteristics

**Figure 1:**
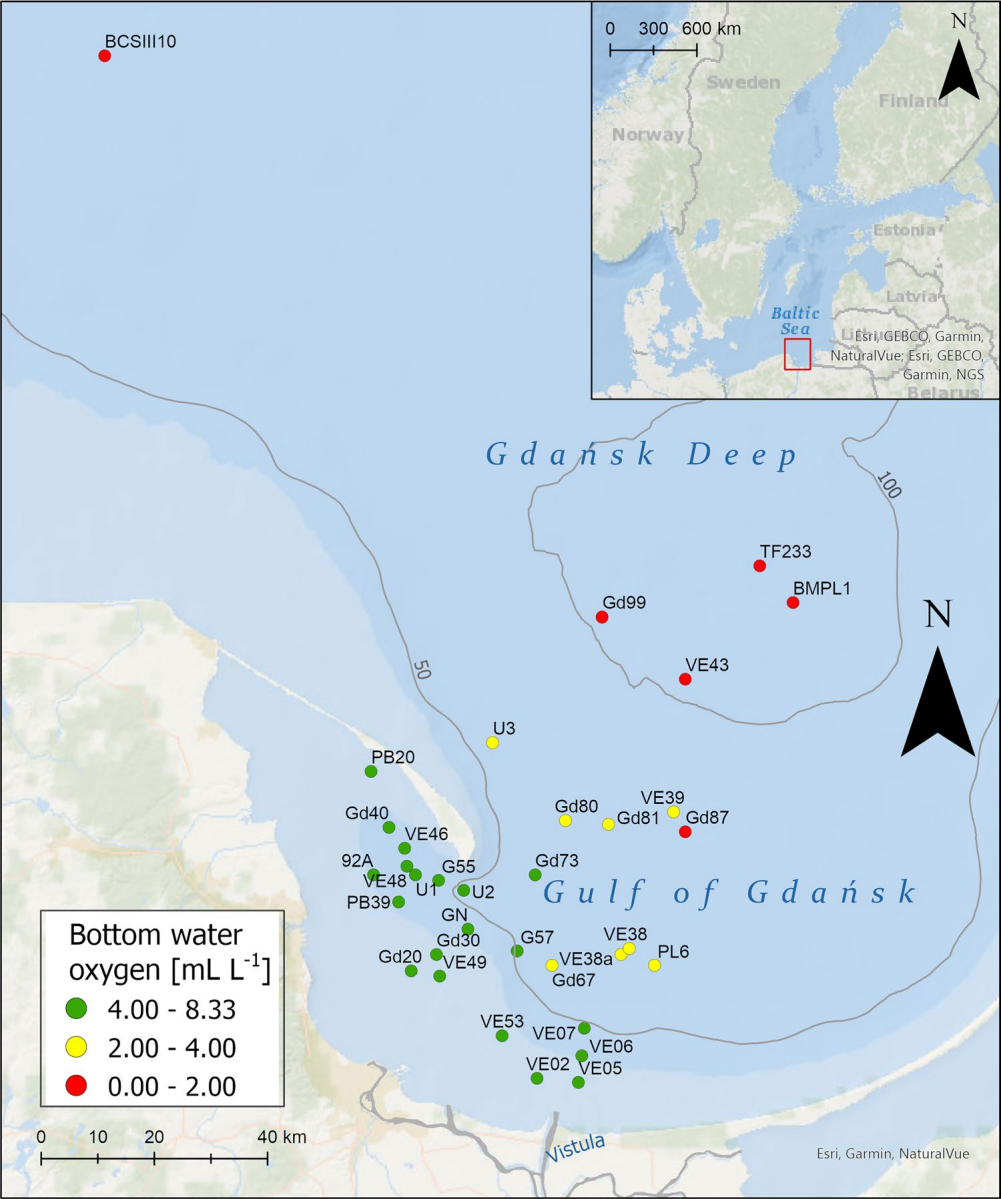
Map of the sampling sites in the Gdańsk Basin.

Three groups of sites were defined based on the oxygen condition: normoxia with good oxygen conditions (DO ≥ 4); reduced of oxygen conditions (DO 2–4); and hypoxia (DO < 2). Dissolved oxygen in bottom water ranged from 4.11 to 8.41 mL L^−1^ in the group of normoxic sites, from 2.55 to 3.91 mL L^−1^ at sites with reduced oxygen conditions, and from 0.63 to 1.59 mL L^−1^ at hypoxic sites (Table 1). The salinity of bottom water at the analyzed sites (Fig. 1) was typical of the Gdańsk Basin and increased with depth from 6.7 (20 m) to a maximum of 13.8 (107 m; Table 1).

**Table 1:** Ranges of depth and near-bottom water parameters at the sampling sites in the oxygen groups: DO ≥ 4 (DO ≥ 4.0 mL L^−1^), DO 2–4 (DO 2.0–4.0 mL L^−1^), DO < 2 (DO < 2.0 mL L^−1^).

	DO ≥ 4	DO 2–4	DO < 2
Number of sites	20	8	6
Depth [m]	20–73	67–84	87–107
Salinity	6.7–10.3	9.2–12.7	10.4–13.8
Temperature [°C]	1.8–16.7	2.0–7.4	5.1–7.5
Oxygen [mL L^−1^]	4.11–8.41	2.55–3.91	0.33–1.59

### Taxonomic richness and biomass of macrofauna

**Figure 2:**
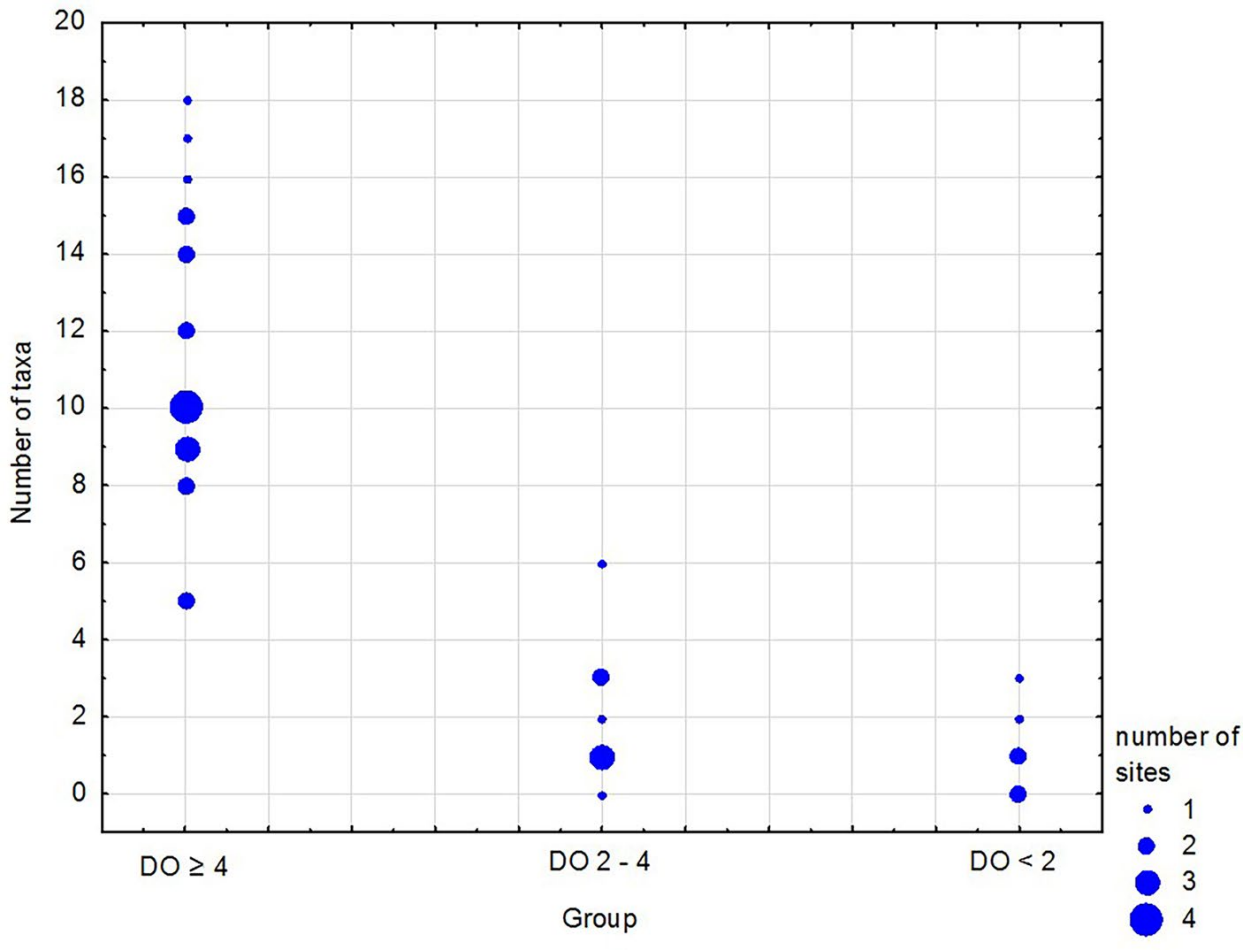
Number of taxa at the sampling sites in the oxygen groups. See Table 1 for group characteristics.

A total of 34 species or higher taxa were identified at all the surveyed sites. The highest number of taxa (18) was recorded at a depth of 35 m (site GN). In general, the number of taxa per site decreased with increasing depth and deterioration of oxygen conditions (Fig. 2). The fewest or complete absence of macrozoobenthos taxa was recorded in the two groups of sites below the halocline, statistically significantly less than in normoxia group (Kruskal–Wallis test *p* < 0.0001, H = 23.698, df = 2; post-hoc test *p* < 0.001). Between five and 18 taxa (on average 11) were present at individual sites in the shallowest part with DO ≥ 4.0 mL L^−1^, and all taxa recorded during this research were present at the sites in this group. From zero to six species were present at the sites where oxygen concentration was lower than that under normoxia and under hypoxia, with a total of nine and four taxa recorded, respectively. No macrofauna was recorded at three sites, and only the polychaete *Bylgides sarsi* was present at five other sites.

**Figure 3:**
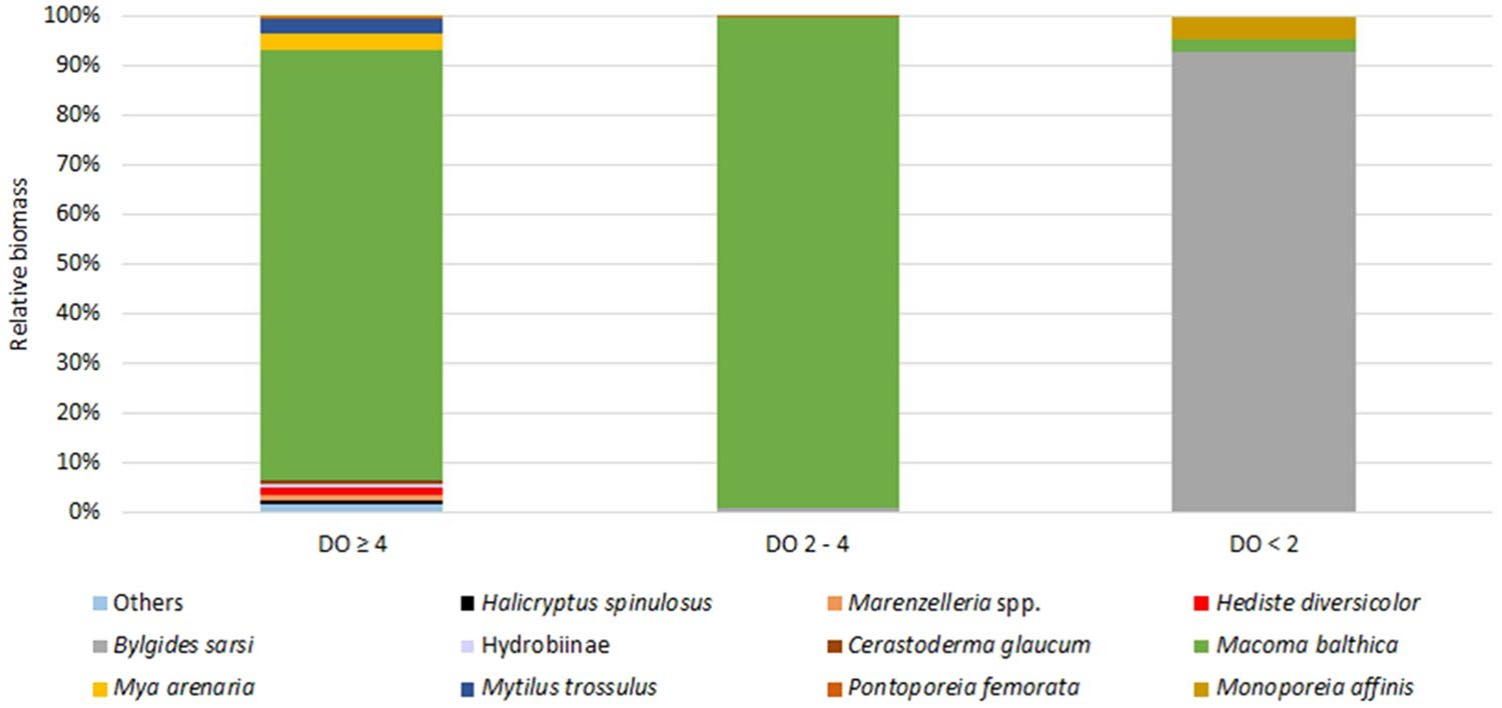
Macrozoobenthos relative biomass in each group of the sites. Others—taxa with biomass < 1% in any group. See Table 1 for group characteristics.

Under good oxygen conditions, the biomass at individual sites ranged from 67.4 g m^−2^ (site PB39) to 546.3 g m^−2^ (site VE48) and averaged 188.2 g m^−2^. The bivalve *Macoma balthica* accounted for the largest proportion of biomass (Fig. 3), averaging 163.3 g m^−2^ (85%)*.* Eight other taxa contributed > 1% to the biomass: the priapulid worm *Halicryptus spinulosus*, polychaetes *Marenzelleria* spp., *Hediste diversicolor*, bivalves *Cerastoderma glaucum, Mya arenaria, Mytilus trossulus*, gastropods from Hydrobiinae, and the crustacean *Pontoporeia femorata* (Fig. 3). Below 80 m the biomass was significantly lower than that under good aerobic conditions, averaging 34.8 g m^−2^ under deteriorated oxygen conditions and was drastically lower under hypoxia (0.3 g m^−2^). There was significant difference in community structure (characterized by the biomass of taxa) between all groups (ANOSIM global test R = 0.704, *p* = 0.001) and in the pairwise test between the normoxia group and the reduced oxygen conditions group (ANOSIM pairwise test R = 0.833, *p* = 0.001), as well as between the normoxic group and the hypoxic group (pairwise test R = 1, *p* = 0.001).

The highest average similarity (SIMPER) between the sites in each group (56%) was observed under good oxygen conditions, where the taxa responsible for the similarity between the sites were *M. balthica* (82%) and, to a much lesser extent, *Marenzelleri*a spp. (4%), *M. arenaria* (3%) and *H. diversicolor* (2%). The internal similarity at sites with a DO range of 2.0–4.0 mL L^−1^ was 24%, with 98% of this similarity attributed to the presence of *B. sarsi*. Under hypoxia, the similarity was 24%, which was solely due to the presence of *B. sarsi.* The average difference between the group of normoxic sites and other groups was higher than 88%.

### Biological traits and functional diversity

The number of represented modalities decreased with the deterioration of oxygen conditions. At the sites with normoxia, all 66 analyzed modalities of the 16 biological traits were represented by macrozoobenthos (Table 2). Between 50 and 64 categories were represented at individual sites under good oxygen conditions. Fewer, 53 modalities (80%), were recorded under reduced oxygen conditions. Between 19 and 35 modalities were represented at sites with DO < 2.0 mL L^−1^, where fauna was present. In the group of hypoxic sites, none of the modalities were represented (present) in more than 60% of the samples (grabs) from the sites in this group. At the same time, very rare and rare modalities (represented by one or up to two taxa, respectively) were recorded in all groups. Under normoxia, five modalities were identified as very rare and four as rare. At the sites from the group under reduced oxygen conditions, nine modalities were rare, and 13 modalities were represented by one taxon. At the same time, of all 42 modalities represented under hypoxia, 18 modalities were represented by only one taxon, and 17 were represented by two taxa.

**Table 2:**
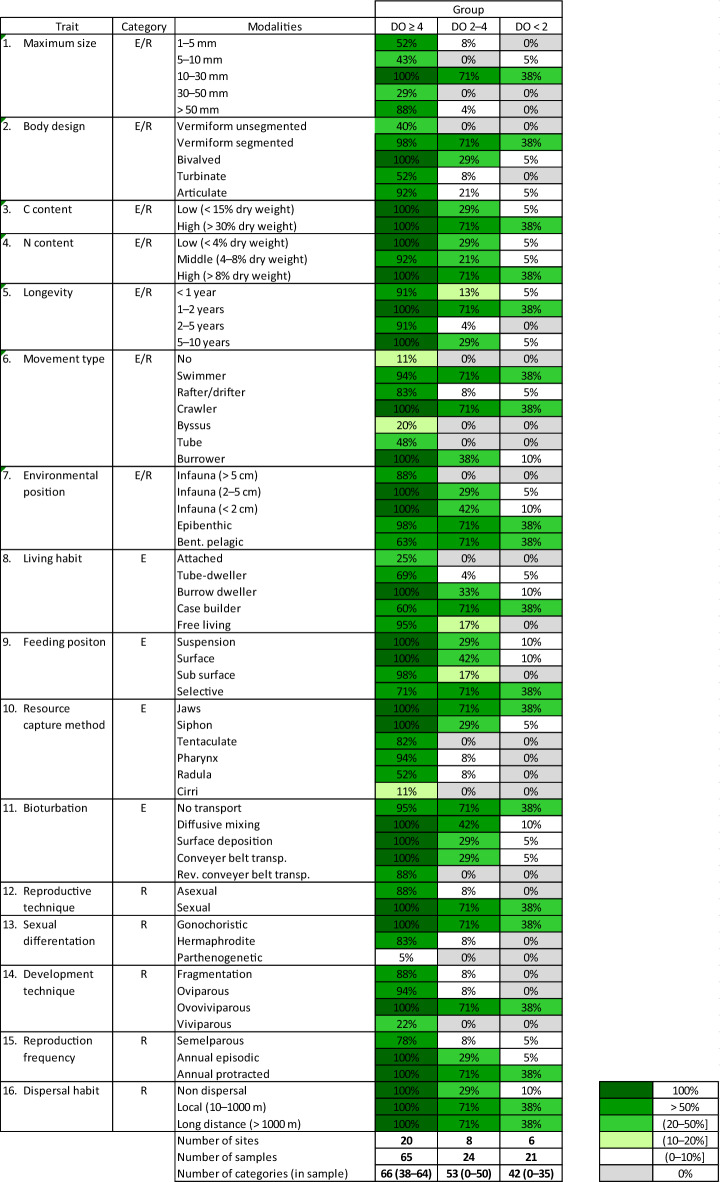
Frequency of trait modalities in samples from groups of sites based on taxa composition. Trait categories: E, effect trait; R, response trait, based on de Juan et al.^35^. See Table 1 for group characteristics.

**Figure 4:**
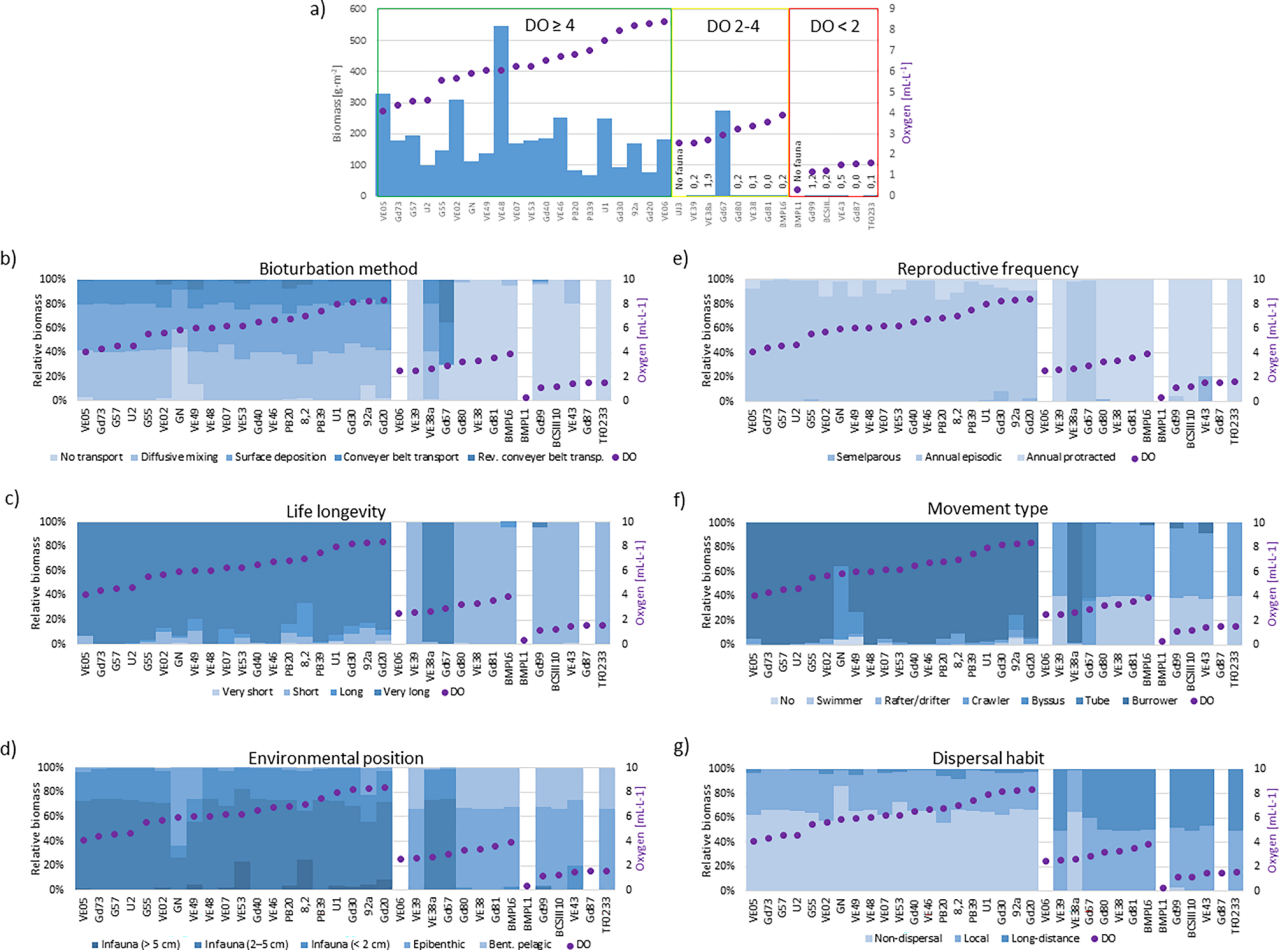
(**a**) Fauna biomass (total biomass value labels are given when biomass < 2 g m^−2^) and percentage of trait modalities at different sites; (**b**) bioturbation; (**c**) longevity; (**d**) Environmental position; (**e**) reproduction frequency; (**f**) movement type; (**g**) dispersal habit along with bottom water oxygen concentration [mL L^−1^] at sampling sites.

The macrofauna biomass and selected biological traits, as well as dissolved oxygen concentration at each site, are presented in the graphs (Fig. 4). The percentage of individual biological traits in the total macrofauna biomass varied at the sites characterized by good oxygen conditions. Traits reflecting the impact of organisms on sediment structure and nutrient concentrations in pore water (Bioturbation method, Environmental position) indicate a diverse and strong impact under normoxic conditions, but little or no effect under other conditions (Fig. 4b,d). In the case of another effect trait, Longevity, very long-lived organisms dominated at normoxic sites, while short-lived ones dominated in the other groups. As for traits relevant to species dispersal (Fig. 4e–g), such as Reproduction frequency, which is important for colonization of new areas, annual episodic and annual protracted predominated under good aerobic conditions. The latter dominated under lower oxygen conditions (Fig. 4e). Under good oxygen conditions, more than half of the biomass consisted of non-dispersal and local organisms, dispersing within a distance of 10–100 m (Fig. 4g). Both groups of deteriorated oxygen conditions included local or long-distance migrating organisms.

**Figure 5:**
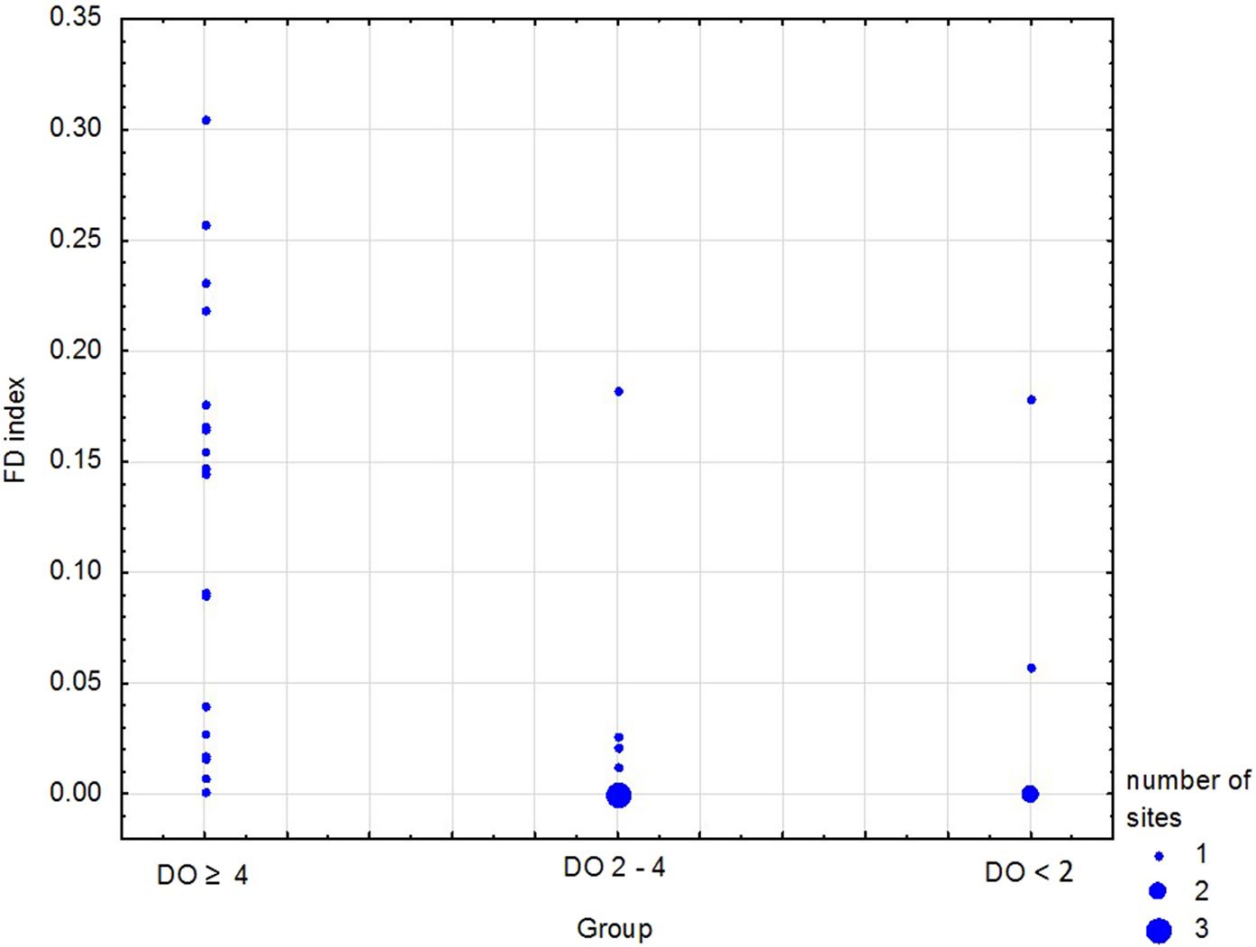
Index of functional diversity (FD) of benthic fauna at the sampling sites in the oxygen groups. See Table 1 for group characteristics.

The FD index presented in Fig. 5 for the sampling sites is a measure of dissimilarity between taxa based on traits and relative biomass of taxa in the community.

The values and variability of FD index were higher in good oxygen group compared to the other groups. The median FD index at the sites under normoxia was 0.12 ± 0.10, which was an order of magnitude higher than in the group of sites with reduced oxygen conditions (0.03 ± 0.07) and the group of hypoxic sites (0.06 ± 0.07; Fig. 5).

## Discussion

In the soft sediments of Gdańsk Basin, oxygen is the main factor affecting the structure and biomass of benthic communities. Its deterioration follows a gradient of increasing depth, as a consequence of decreasing sediment grain size and increasing organic matter content. Additionally, toxic hydrogen sulfide is released during decomposition processes. Under good oxygen conditions, diverse and abundant benthic fauna communities occur on all types of sediments—from coarse-grained sands with low organic matter content to silts with much higher organic matter content^71^.

Zoobenthic communities, representing the largest number of biological traits, were observed in the zone above the halocline with good oxygen conditions in bottom waters. All 66 analyzed modalities were observed above the halocline. Törnroos et al.^39^ reported a comparable richness of modalities (common in theirs and our study) of biological traits for communities from the southern Baltic Sea. Taxonomic richness in the Baltic Sea decreases due to the salinity gradient^52^, which leads to an overall reduction in number of trait categories represented, but functional richness remains relatively high even at the lowest level of taxon richness^39^. In the deterioration of oxygen conditions in the Gdańsk Basin, we observed a 34% decline in modalities. In all groups, we observed very rare and rare modalities (i.e., represented by one or a maximum of two taxa, respectively). While in the zone characterized by good oxygen conditions, they accounted for only about 14% of all modalities represented, at oxygen-depleted sites they accounted for 58%, and under hypoxia as much as 83% of the modalities present were represented by up to two taxa. According to the redundancy hypothesis^53^, there is a minimum level of diversity to ensure the functioning of an ecosystem. In addition to those providing the minimum, the remaining taxa constitute a kind of protection in case of possible disturbances^53^, such as periodic oxygen deficiency at the bottom. In the case of the southern Baltic deep water region, there is no such protection, as only single taxa with extremely low biomass occur there.

In addition to the richness of biological traits, the FD index is also important when describing functional diversity. In our case, the FD index is a proxy for the dissimilarity between traits, which includes proportion of a biomass in the community. The highest FD index (maximum of 0.31) was observed at the normoxic sites, with a median value of 0.12, which is five times higher compared to the depleted sites. Due to the low biomass of macrofauna below the halocline, the lack of diversification of traits between the taxa forming a community, and the strong dominance of a single taxon at the depleted and hypoxic sites, the median FD index was very low and dropped to 0 at the sites with one taxon. All the obtained values were lower than the results obtained in the western Baltic Sea (range 0.12–0.56), where the recorded number of taxa was significantly higher for most of the study period^48^. Studies of benthic communities at the soft seabed of the Baltic Sea showed that FD followed a decline in taxonomic richness^39^. The relatively low FD values observed at the normoxic sites are due to high average similarity of the benthic communities of soft-bottom habitats due to the strong dominance (> 85%) of *M. balthica* in the total biomass, which is typical in Gdańsk Basin^26,28,58^. Research in the Aegean Sea has also shown that the FD index (maximum of 0.30) is higher in areas with diverse habitats^54^.

Hypoxia alters ecosystem functioning by reducing the number of species, density or biomass, or even the total loss of zoobenthos^1,16,29^. With the loss of taxa, the traits they represent are also lost to the ecosystem, and individual traits reflect the functions performed by organisms in a given ecosystem. For example, the maximum size of adult specimens relates to i.a. trophic relationships, which in turn affects the circulation of matter in an ecosystem^55^. The lifespan of organisms is considered a proxy for energy fixation, turnover and production rate^56^. In the Gulf of Gdańsk, organisms ranging from very small to the largest were recorded under normoxic conditions, while only species growing to intermediate size as adult maxima, were present under deteriorated oxygen conditions and hypoxia. In addition, the frequency of organisms in all lifespan modalities is lower in oxygen-deficient regions. The obtained results corroborate the work by Pacheco et al.^30,57^, who recorded large and medium-sized organisms as well as those with a lifespan of up to 12 year in shallow-water (15 m) habitats in Mejillones Bay (northern Chile). While at a depth of 50 m, in an oxygen-deficient region, only small (but not the smallest) taxa, with a lifespan of several years were observed. In the brackish waters of the Baltic Sea, oxygen-deficient regions are dominated by small species that burrow only in the surface layer of sediments, including zones where large, perennial, deep-burrowing taxa were previously observed^58,59^. Among the taxa recorded in the Gulf of Gdańsk, long-lived taxa include bivalves. Other taxa live from several months (i.a. small polychaetes and crustaceans) to several years (e.g., priapulid worms, large polychaetes). The size of detritivores determines the size of the food particles they choose^60,61^ and, at the same time, along with their environmental position, their vulnerability to predator attacks. Of the organisms recorded in this study, the largest (> 50 mm) were adults of selected polychaete species, e.g., *Marenzelleria* spp., the bivalve mollusk *M. arenaria* and the crustacean *S. entomon*. They provide a food base for numerous species of fish^62,63^ and diving birds^64,65^. The smallest specimens were those of taxa recorded only under normoxia, such as *Jaera* spp., Hydrobiinae and *Fabricia stellaris*. These taxa constitute a food base for benthic invertebrates^66^ and fish^67^, including juveniles. Consequently, oxygen deficiency in the seabed zone causes changes and disruptions in the circulation of matter, while the reduction in the size and complexity of benthic food webs alters and disrupts energy pathways^1,20^.

With regard to ecosystem functioning, a separate group consists of traits directly affecting the biotope, categorized by de Juan et al.^35^ as effect traits. Among traits related to the role played by organisms directly in the sediment matrix (i.a. environmental position and type of bioturbation), all functional categories were recorded only under good oxic conditions. As aerobic conditions deteriorate, species that are important in organic matter processing—penetrating deep into the sediment, forming complex biogenic structures in the sediment, feeding on subsurface detritus and carrying particles from deeper layers of the sediment to its surface (matter conveyors)—disappear. While the presence of infauna in the upper 2 cm of the sediment was still detectable under deteriorated oxygen conditions, only epifaunal and bentho-pelagic taxa, feeding on detritus on the sediment surface, as well as crawling and swimming organisms were found under hypoxic conditions. These results correspond with previous observations by other authors indicating that as oxygen conditions deteriorate, taxa that penetrate deep layers of the sediment, forming biogenic structures there and feeding on subsurface detritus, as well as sediment conveyors, are lost first^29,30,48,68^. Even short-term, but often recurring hypoxia leads to a significant reduction in the bioturbation potential of benthic communities through, among other things, removal of large deep burrowing individuals^47,69^. Under oxic conditions, fauna has a significant impact on solute fluxes^50^ and can increase the removal of phosphate and silicate from sediments into the bottom water^70^. Most of the species recorded in our study are associated with the surface layer of the sediment, but some taxa, such as *M. balthica*, *M. arenaria* and polychaetes *Marenzelleria* spp., are able to burrow deep into the sediment^71^, given that deep burrowers are adults and the largest individuals. Most individuals, including juveniles, occur on the surface layer of the sediment^71,72^. Organisms responsible for transporting and mixing sediment particles contribute, among other things, to the transfer of fresh organic matter from the surface into deeper layers of the sediment^73^, and by oxygenating interstitial water, among other things, increase the rate of mineralization of organic matter in the sediments^74,75^. Moreover, through the disruption of the structure of macrofauna communities and species burial depth, oxygen deficiency affects biogeochemical processes^47,69,70,76,77^.

Persistent hypoxia leads to a decline in the biodiversity of macrofauna, while the possibility of returning to a state enabling proper functioning (recovery) is facilitated by the presence of undisturbed communities in the immediate vicinity, i.e., the recovery potential of the ecosystem after hypoxia^78^. The taxonomic richness and density of the macrofauna occurring in the zone above the halocline constitutes a potential reserve for the recovery of benthic communities when oxygen conditions improve in deeper regions. According to the category of response traits^35^ (Table 2), reproduction, locomotion and dispersal traits are essentials for recovery. Reproductive traits, such as methods and frequency of reproduction, are important for, inter alia, the possibility of restoring benthic communities in post-disturbance areas^48,79^. Our study shows that under normoxia, organisms reproduce both sexually and asexually. At sites belonging to other groups, almost exclusively sexually reproducing species were observed, in contrast to the results reported from northern Chile^30,80^, where only asexually reproducing polychaetes were observed in oxygen-deficient zones. Sexual reproduction provides genetic variation and thus increases a system resilience and recovery potential after disturbance^81^, but there is no external supply of benthic propagule into the deep habitat of the Chilean coast^30^. The presence of organisms with a long reproductive season in the ecosystem is also important for the process of succession after a disturbance, as they are more likely to colonize the region if conditions improve^82^. Organisms representing all reproduction frequencies were recorded above the halocline, including numerous taxa that reproduce for several months of the year, such as *M. balthica.* While macrozoobenthos was present in the area of the Gdańsk Deep and its slope in the past^58^, not only has no macrozoobenthos been observed in recent years, but hydrogen sulfide may be present in the water above the sediment up to nine months of the year^83^. We recorded two taxa in this area—polychaetes *B. sarsi* and juveniles (length < 5 mm) of *M. balthica*. While *B. sarsi* quickly colonizes areas as soon as oxygen conditions improve there^48,84^, *M*. *balthica*, on the other hand, takes several months to grow to a few millimeters, so its individuals are indicators of good oxygen conditions for at least several months before harvesting. The latter species reproduces in the southern Baltic Sea in spring and autumn^85^. The presence of both taxa is due to a series of major water inflows into the Baltic Sea that occurred in 2014–2015^86^. Macrozoobenthos was no longer recorded in samples collected from the Gdańsk Deep in 2016.

Regeneration is also determined by the available pool of mobile colonizers in undisturbed regions^82^. The mode of locomotion and dispersal in the environment affects the production or dispersal potential of organisms as well as their colonization potential^37,48,56^. Under fully oxygenated conditions, species with diverse (including long-distance) dispersal habits are found. Experimental field studies^87^ conducted in shallow water areas showed that the first colonizers of the experimental area were adult immigrants settling at the top sediment layer. While adult crustaceans, gastropods or polychaetes, due to their vertical migration, can successfully colonize a previously disturbed area^47^, adult *M. balthica* or *M. arenaria* are not be expected there. According to Beukema et al.^88^, colonization of post-hypoxic areas by macrofauna is a complex process—most taxa colonize through settling pelagic larvae or juveniles, as in the case of taxa with simple development, but colonization by adults also occurs. On the other hand, research conducted in the environment after natural and human-induced improvement of oxygen conditions in, among others, the fjords of western Sweden indicated colonization by settling larvae, mobile bentho-pelagic organisms and epifauna^78,89^. Research on succession after the cessation of oxygen deficiency unanimously indicates that an increase in species richness and density occurs rapidly (from several summer months to a year), but biomass comparable to that in undisturbed areas and the occurrence of very large individuals was recorded only after several years^78,90^.

The rate of recolonization is also determined by spatial scale^82^, the impact of other disturbances such as excess organic matter or recurrent oxygen deficiency^69^. However, the return of large bivalve or shellfish individuals, including long-lived sedentary species, depends on long-term improvement in oxygen conditions. Although it is potentially possible for organisms to form benthic communities once adverse oxygen conditions cease^91^, this involves a change in the structure of the entire community in favor of taxa that have adaptations to conditions of reduced oxygen concentration and hydrogen sulfide^92^. In the zone above the halocline, which allows the recovery of macrofauna in deeper regions, taxa with high tolerance to oxygen deficiency were observed, including *H. spinulosus*, *Marenzelleria* spp., *S. entomon* and *M. balthica*, provided that these are not long-term events^22,45,93,94^.

Although ecosystem resistance depends, among other factors, on the composition of benthic communities and their resilience^95,96^, ecosystem recovery depends primarily on the improvement of oxygen conditions. In studies of the recovery potential of macrozoobenthos, it is also necessary to consider other biological response traits that enable the survival under these adverse conditions (including having large energy reserves that can be used in anaerobic metabolism) and the availability of planktic larvae.

As oxygen conditions deteriorated, the biomass, diversity and functionality of the macrozoobenthos declined markedly. Although a few taxa observed under hypoxia, their biomass was extremely low. Both very small and large organisms penetrating deep into the sediments disappear in depleted conditions. In the area under hypoxic conditions, we observe only small, mobile organisms with high turnover of energy and less stable storage of carbon and nitrogen compared to large, long-lived fauna. On the positive site, they can spread quickly when oxygen conditions improve. However, these species do not form complex structures in the sediments and do not cause bioturbation and bioirrigation in deeper sediment layers. Thus, animals are not able to enhance ecosystem functions as production, decomposition or nutrient cycling. Traits important for species dispersal and the presence of taxa temporarily resistant to hypoxia indicate that benthic communities above the halocline may be a potential source of organisms for recolonization of deeper areas in the southern Baltic Sea.

## Methods

### Study area

The research was carried out in the Gdańsk Basin, in the southern Baltic Sea. The basin consists of the shallower Gulf of Gdańsk in the south and the adjacent Gdańsk Deep, with a maximum depth of 114 m. A seasonal thermocline occurs in this area at a depth of about 30–40 m, and a halocline is usually observed at a depth of about 60–80 m. The bottom sediments of the Gdańsk Basin have a complex structure—sands or mixed sandy sediments in the shallow waters, silts in the central part of the Gulf Gdańsk, and clayey-silty sediments in the Gdańsk Deep^97^. The Gdańsk Basin is strongly impacted by the Vistula River, which is a great source of nutrients and organic matter to the system^98–100^. In general, oxygen conditions in the depth zones are typical for this basin: those above the halocline are good throughout the year^26^. Episodic oxygen deficiencies are observed in the zone below the halocline^26,28,99,101^. In the Gdańsk Deep, on the other hand, starting from its slope (at a depth of approximately 80 m), oxygen deficiencies occur regularly, even for several months a year^3,102,103^. Anoxic conditions with hydrogen sulfide in the bottom water were observed in the Gdańsk Deep area almost every year in last two decades^3^.

### Sampling and environmental data

Thirty-four sampling sites were located in a gradient of oxygen conditions in the bottom zone in the depth range of 20–107 m (Fig. 1). Samples were collected during several cruises between 2009 and 2016 (Supplementary Table S1). Data for two sites collected during HELCOM monitoring in 2015 were obtained from ICES Database DOME^104^. Water parameters were analyzed approximately 0.5 m above the seabed. Bottom water temperature and salinity were measured instantaneously using a Seabird CTD system or a multi-parameter 340i WTW Gmb meter. Dissolved oxygen (DO) concentration was measured using Winkler titration or a Seabird SBE43 oxygen sensor (Supplementary Table S1).

### Biological data analysis

Samples of macrozoobenthos for species and functional diversity analysis were collected using a standard method with a 0.1 m^2^ Van Veen grab sampler (1 mm sieve), with 1–5 replicates collected at each site. The residual material remaining on the sieve was preserved with 4% formaldehyde solution. Organisms were identified to the lowest taxonomic unit and counted, and their formalin wet weight was determined to the nearest 0.1 mg. Oligochaeta, polychaetes *Marenzelleria*, mud snails Hydrobiinae, crustaceans *Jaera*, juveniles of *Idotea* and larvae of Chironomidae were not identified to the species level.

Hypoxic conditions are understood differently by different authors and the effects of oxygen deficiency on organisms vary for different species or even life stages of specific species^92,105^. We have adopted the definition of hypoxia from Diaz and Rosenberg^1^ as oxygen concentrations below 2 mL L^−1^, while anoxic conditions refer to the lack of oxygen in the water. Lower oxygen levels become harmful not only when there is hypoxia in the environment, but also when reduced oxygen conditions cause behavioral and physiological responses, such as reduced growth, loss of reproductive capacity, mortality, etc.^15,20,91,106^. To determine the macrofauna characteristics in relation to oxygen conditions, the sites were divided into three groups based on the concentration of dissolved oxygen in bottom water at the surveyed sites during sampling: normoxia DO ≥ 4.0 mL L^−1^; deterioration of oxygen conditions DO 2.0–3.9 mL L^−1^; and hypoxia DO < 2.0 mL L^−1^ (Supplementary Table S1).

### BTA—biological traits analysis

Biological traits analysis (BTA) was used to determine the functional diversity of macrofauna. Each taxon was analyzed and classified based on 16 selected biological traits (Table 2), within which 66 modalities (categories) were distinguished. The classification was based on information available in peer-reviewed scientific literature and databases (i.a.^48,107–109^; Supplementary Table S2). Biological traits modalities were assigned to the lowest possible taxonomic level. The fuzzy coding approach^110^ was performed based on the affinity of each taxon to the trait. It was scored from 0 to 1, where 0 indicates no affinity of a taxon to a trait category and 1 indicates absolute affinity^38^.

The number of taxa representing particular modality in every oxic group was counted. Trait modalities were considered rare if they were represented by two taxa in a given group, while modalities that were represented by one taxon were classified as very rare. The frequency of each functional modality in the groups of sites was analyzed based on the presence/absence of each modality in individual samples (grabs).

### FD—Functional Diversity index

Rao's quadratic entropy (generalization of Simpson’s diversity index) was used as a measure of functional diversity (FD) of community. The FD index was determined in the free macro “FunctDiv.xls”^111^, based on the matrices: taxa by traits modalities and biomass of taxa.

For each trait, FD is a measure of distances between pairs of taxa in terms of trait modalities they represent in the entire community, expressed by relative biomass at given site. The FD index is the average value of FD calculated for individual traits, with the range from 0 to 1. If there is a single taxon or all taxa in the community have exactly the same traits, the FD has a minimum value of 0. If every pair of species in the community is completely different in terms of traits, the index reaches the highest value and is equal Simpson’s diversity index (expressed as 1 minus Simpson’s index of dominance).

### Statistical analysis

Analysis of Similarities (ANOSIM) was used to determine differences in the taxonomic composition of benthic invertebrate communities and macrofauna biomass at the sampling sites, followed by SIMPER similarity analysis (Similarity Percentages) to assess which taxa were responsible for an observed difference between groups. Prior to similarity analysis, the biomass of individual taxa was transformed (√)^112^. The nonparametric multivariate Kruskal–Wallis test was used along with post-hoc pairwise comparisons to compare the number of taxa between the sites from the oxygen groups. The analyses were performed using Dell Statistica 13.1 and Primer 7 (PRIMER-E Ltd).

Spatial representation of the results on maps was performed in ArcGIS Pro 2.6.0 ESRI Inc.

### Supplementary Information

Supplementary Tables.

## Data Availability

The datasets generated and/or analyzed during the study are available from the corresponding author on reasonable request.
